# Relationship of Arterial Compliance and Blood Pressure with Microalbuminuria and Mildly Decreased Glomerular Filtration Rate: A Chinese Community-Based Analysis

**DOI:** 10.1371/journal.pone.0101013

**Published:** 2014-06-25

**Authors:** Shihui Fu, Yuqing Sun, Leiming Luo, Ping Ye

**Affiliations:** 1 Department of Cardiology and Hainan Branch, Chinese People's Liberation Army General Hospital, Beijing, China; 2 Department of Geriatric Cardiology, Chinese People's Liberation Army General Hospital, Beijing, China; Emory University, United States of America

## Abstract

**Objective:**

This analysis is designed to determine the prevalence of microalbuminuria (MAU) and mildly decreased glomerular filtration rate (GFR); to investigate the association of augmentation index (AIx), central blood pressure (cBP) and peripheral blood pressure (pBP) with MAU and mildly decreased GFR; and to compare the association strength of cBP and pBP with MAU and mildly decreased GFR.

**Methods:**

This community-based analysis included 2071 Chinese residents. Urine albumin-to-creatinine ratio (UACR), GFR, and pulse wave measurements were performed. UACR of 30–299 mg/g and GFR of 60–89 ml/min/1.73 m^2^ were identified as MAU and mildly decreased GFR.

**Results:**

The prevalence of MAU and mildly decreased GFR was 21.3% and 33.2%. The AIx, cBP and pBP were significantly higher in participants with MAU compared with those without MAU, and in participants with mildly decreased GFR compared with those without mildly decreased GFR (all P<0.001). After participants were categorized into four subgroups based on the presence or absence of MAU and mildly decreased GFR, Aix, cBP and pBP progressively increased from the subgroup without both of MAU and mildly decreased GFR to the subgroups with either one of them, and arrived at top in the subgroup with both of them (all P<0.001). Compared with the reference category without MAU and mildly decreased GFR, the odd ratio values significantly increased from the category with either one of MAU and mildly decreased GFR to the category with both of them (all P<0.001). The AIx, cBP and pBP were all independently associated with MAU and mildly decreased GFR after full adjustment (all P<0.05), and the association strength of MAU and mildly decreased GFR with cBP was similar to those with pBP.

**Conclusions:**

In Chinese community-dwelling population, there was a high prevalence of MAU and mildly decreased GFR. The AIx, cBP and pBP were all independently associated with MAU and mildly decreased GFR; meanwhile, cBP did not exhibit stronger association with MAU and mildly decreased GFR compared with pBP.

## Introduction

Recent interest has been aroused in the relationship of renal demage with arterial compliance evaluated by augmentation index (AIx) and blood pressure including central blood pressure (cBP) and peripheral blood pressure (pBP). Several studies have investigated the association of AIx, cBP and pBP with glomerular filtration rate (GFR) and urine albumin-to-creatinine ratio (UACR) as a continuous variable, but they obtained the inconsistent results [1]–[10]. Meanwhile, although other studies have analyzed the relationship of AIx, cBP and pBP with chronic kidney disease (CKD), macroabuminuria and end-stage renal disease (ESRD) [3], [11]–[13], the association of AIx, cBP and pBP with microalbuminuria (MAU) and mildly decreased GFR has not been fully explored, and to date the available data have been unable to give a definite conclusion as well [3], [6], [9], [14], [15]. In particular, most studies have paid attention to the correlation of AIx, cBP and pBP with CKD and ESRD, but their relation to the early stage of renal function, i.e., mildly decreased GFR, is a relatively neglected topic [15]. The study by Dr. Ashish Upadhyay has shown that MAU was associated with cBP rather than AIx [3]. However, Dr. Costas Tsioufis has suggested that MAU was closely related to AIx [6]. The ethicty is an important factor that can affect the relationship of AIx, cBP and pBP with MA and mildly decreased GFR, and most previous studies have focused on the westerners rather than Chinese community-dwelling residents. To our knowledge, there is almost no community-based study relating the AIx, cBP and pBP to MAU and mildly decreased GFR in China. Thus, it is necessary to conduct a large community-based study in China to analyze the relationship of AIx, cBP and pBP with MAU and mildly decreased GFR.

In addition, scarce studies have compared the association strength of cBP and pBP with MAU and mildly decreased GFR. Dr. Kang-Ling Wang has demonstrated that cBP was more strongly related to GFR compared with pBP [16]. Dr. Ulf Neisius has indicated that cBP and pBP had equal association with UACR [17]. The lastest study from Dr. Anna Oliveras has realized that neither cBP nor pBP was superior to each other in their association with MAU [9]. Thus, the performance of cBP and pBP in the identification of MAU and mildly decreased GFR need to be observed in more large-scale studies, and it remains to be seen whether cBP is superior in identifying MAU and mildly decreased GFR.

Therefore, in this analysis we investigated the Chinese community-dwelling population: 1) to determine the prevalence of MAU and mildly decreased GFR; 2) to investigate the association of AIx, cBP and pBP with MAU and mildly decreased GFR; and 3) to compare the association strength of cBP and pBP with MAU and mildly decreased GFR.

## Materials and Methods

### Study population

This community-based analysis enrolled 2071 permanent residents in Beijing, China. All participants were of Han origin, over 18 years of age, all of whom were involved in the large health survey from May 2007 to July 2009. A stratified cluster sampling design was used in this survey, the purpose of which was to observe the healthy quality of Beijing adults. In the first stage of sampling, 3 districts (Fengtai, Shijingshan, and Daxing) were selected from 18 districts in Beijing. In the second stage of sampling, four communities were selected from these districts. In the third stage of sampling, the participants were selected from these communities. Subjects with macroalbuminuria (13 subjects) and GFR <60 ml/min/1.73 m^2^ (18 subjects) were excluded because they were unsuitable for our analysis objective. Thus, 2040 participants were eligible for analysis. This study was in compliance with the Helsinki Declaration. It was approved by the Ethics Committee of Chinese People’s Liberation Army General Hospital (Beijing, China). All patients provided written informed consent to be included in the analysis.

### Physical examination

Weight was measured on a digital scale while subjects wore light clothing with no shoes. Height was determined using a wall-mounted measuring tape, subjects standing with no shoes. Body mass index was calculated as weight (kg) divided by height squared (m^2^). Waist circumstance was measured in standing subjects using a flexible tape, midway between the lowest rib and the iliac crest. Brachial blood pressure was obtained by mercury cuff sphygmomanometer (Yuwell medical equipment & supply Co., Ltd., Jiangsu, China) in a seated position after 5 minutes of rest. Blood pressure was measured in duplicate and averaged. The sequential measurement was in the same arm. In accordance with the current guidelines, the variability between the two readings was allowed to be less than or equal to 5 mmHg. Subjects who had differences greater than 5 mmHg were asked to be measured again after 30 minutes. If the differences were still greater than 5 mmHg, the additional readings should be obtained and averaged. The measurement was completed by physicians in Chinese People's Liberation Army General Hospital who were well-trained by the research team.

### Artery pulse wave analysis

Pulse wave analysis was conducted in the morning, in a quiet environment and in the supine position to assess cBP and AIx. The radial artery was gently compressed with the tip of a tonometer at the site of maximal pulsation. The tonometer contains a micromanometer which provides a very accurate recording of the pressure within the artery. The system software was used to calculate an average radial artery waveform, and the corresponding central artery pressure waveform and cBP were generated using a transfer function of the instrument. AIx was defined as the ratio of augmentation to pulse pressure and expressed as a percentage. As there is a linear relationship between heart rate and AIx, the AIx was standardized to a steady heart rate of 75 beats/min. The right radial artery pressure wave form was recorded using HEM-9000AI (Omron Healthcare, Inc., Kyoto, Japan).

### Laboratory test

Venous blood sample was drawn between 8 a.m. and 10 a.m. after an overnight fast of at least 12 hours. For the patients with fasting blood samples obtained, the standard 75 g oral glucose tolerance test was performed two hours after 75 g glucose loading. All blood samples were routinely stored at 4°C, and delivered to the central laboratory in the Department of Biochemistry, Chinese People’s Liberation Army General Hospital, on the same date. The biochemical variables including fasting blood glucose (FBG), postprandial blood glucose (PBG), triglyceride, high-density lipoprotein-cholesterol (HDL-c) and low-density lipoprotein-cholesterol (LDL-c) were measured by a qualified technician using enzymatic assay (Roche Products Ltd., Basel, Switzerland) on a fully automatic biochemical autoanalyzer (COBAS c6000, Roche Products Ltd., Basel, Switzerland). Type 2 diabetes was defined as follows: FBG ≥7.0 mmol/L, PBG ≥11.1 mmol/L, and/or taking a hypoglycemic medication or insulin. Morning spot urine sample (at least 5 mL of midstream urine) was collected to evaluate the levels of albumin and creatinine, and urine albumin was indexed to urine creatinine to account for difference in urine concentrations (UACR). The UACR of 30–299 mg/g was identified as MAU and ≥300 mg/g was identified as macroalbuminuria [18]. Concentration of serum creatinine was measured by enzymatic assay (Roche Diagnostics GmbH) on a Hitachi 7600 autoanalyser (Hitachi, Tokyo, Japan). The GFR was calculated according to the Chinese modified Modification of Diet in Renal Disease (MDRD) equation: 175×serum creatinine (mg/dl)^−1.234^×age (year)^−0.179^×0.79 (if female) [19]. GFR of 60–89 ml/min/1.73 m^2^ was defined as mildly decreased GFR [18].

### Statistical analysis

Continuous variables were expressed using the mean and standard deviation for data exhibiting normal distribution, and the median and interquartile range for non-normally distributed variables. Categorical data were expressed as number and percentage of the total. The population was initially divided into groups with or without MAU, and subsequently into groups with GFR above or below 90 ml/min/1.73 m^2^. Simple difference between two groups was evaluated using the Student’s t-test for continuous variables (normal distribution), Mann–Whitney U test for continuous variables (abnormal distribution), and x^2^ analysis for categorical variables. After participants were categorized into four subgroups based on the presence or absence of MAU and mildly decreased GFR, the difference between four subgroups was examined by Kruskal-Wallis test and the odd ratio (OR) values under each subgroup compared to the reference subgroup without MAU and mildly decreased GFR were obtained by the Multinomial Logistic Regression analysis. Bivariate correlation was assessed by Pearson (normal distribution) and Spearman (abnormal distribution) coefficients. The Binary Logistic Regression analysis was performed using MAU and mildly decreased GFR as dependent variables and AIx, cBP and pBP as identifiers following three models. In the first model without adjustment, in the second model to include age and gender as adjustment variables, and finally in the third model using age, gender, smoking, coronary artery disease, type 2 diabetes, body mass index, waist circumstance, heart rate, FBG, PBG, triglyceride, HDL-c, LDL-c, angiotensin converting enzyme inhibitor/angiotensin receptor blocker, and statins use as potential confounders. Statistical significance was inferred at a 2-tailed P value<0.05. Statistical analyse was conducted using SPSS version 17 software (SPSS, Inc., Chicago, IL, USA).

## Results

The median (range) age of participants in this analysis was 51 (18–90) years and 61.1% were women. The prevalence of MAU and mildly decreased GFR was 21.3% (435 subjects) and 33.2% (677 subjects), respectively. Clinical characteristics of the study population divided into two groups, with and without MAU or mildly decreased GFR, are shown in Table 1. Not only the peripheral systolic blood pressure (pSBP), but also the AIx and central systolic blood pressure (cSBP) were significantly higher in participants with MAU compared with those without MAU, and in participants with mildly decreased GFR compared with those without mildly decreased GFR (all P<0.001).

**Table 1:** Characteristics of study population grouped in patients with and without MAU or mildly decreased GFR.

Variables	UACR			GFR		
	Normal UACR	MAU	P value	Normal GFR	Mildly decreased GFR	P value
	(n = 1605)	(n = 435)		(n = 1363)	(n = 677)	
Main variables						
cSBP (mmHg)	137(123–152)	150(132–169)	<0.001	136(122–152)	145(131–162)	<0.001
AIx (%)	81(73–88)	84(77–91)	<0.001	81(73–88)	83(76–90)	<0.001
pSBP (mmHg)	126(115–140)	135(120–155)	<0.001	125(115–140)	133(120–149)	<0.001
Baseline variables						
Age (year)	50(42–59)	54(45–62)	<0.001	48(38–56)	58(50–66)	<0.001
Males (%)	649(40.4)	145(33.3)	0.007	552(40.5)	242(35.7)	0.038
Smoking (%)	579(36.1)	136(31.3)	0.062	473(34.7)	242(35.7)	0.642
CAD (%)	198(12.3)	65(14.9)	0.150	178(13.1)	85(12.6)	0.749
Type 2 diabetes (%)	103(6.4)	67(15.4)	<0.001	98(7.2)	72(10.6)	0.008
BMI (kg/m^2^)	26.08(23.53–28.72)	27.51(24.53–30.18)	<0.001	26.13(24.42–28.79)	26.84(24.18–29.53)	<0.001
WC (cm)	87(80–94)	90(82–96)	<0.001	87(79–94)	90(83–96)	<0.001
HR (bpm)	75(68–83)	76(68–86)	0.026	76(68–84)	75(68–83)	0.268
FBG (mmol/L)	5.05(4.74–5.45)	5.19(4.82–5.89)	<0.001	5.04(4.72–5.45)	5.19(4.83–5.60)	<0.001
PBG (mmol/L)	5.76(4.71–7.11)	6.43(5.16–8.43)	<0.001	5.74(4.66–7.07)	6.17(5.07–7.97)	<0.001
Triglyceride(mmol/L)	1.29(0.90–1.84)	1.56(1.04–2.26)	<0.001	1.28(0.88–1.86)	1.45(1.04–2.14)	<0.001
HDL-c (mmol/L)	1.44(1.23–1.67)	1.42(1.19–1.65)	0.193	1.43(1.23–1.66)	1.45(1.22–1.71)	0.242
LDL-c (mmol/L)	2.79(2.31–3.35)	2.94(2.46–3.50)	0.001	2.73(2.29–3.32)	2.94(2.48–3.50)	<0.001
ACEI/ARB (%)	18(1.1)	8(1.8)	0.237	12(0.9)	14(2.1)	0.024
Statins (%)	6(0.4)	3(0.7)	0.636	7(0.5)	2(0.3)	0.730
Dependent variables						
UACR (mg/g)	8.64(4.44–14.07)	58.33(40.44–92.64)	<0.001	10.83(5.69–22.78)	12.58(5.47–28.92)	0.094
GFR(ml/min/1.73 m^2^)	96.52(87.79–106.93)	94.33(85.83–106.35)	0.058	102.56(96.18–111.78)	83.73(78.15–87.42)	<0.001

**Abbreviation**: MAU: microalbuminuria; GFR: glomerular filtration rate; UACR: uric albumin-to-creatinine ratio; cSBP: central systolic blood pressure; AIx: augmentation index; pSBP: peripheral systolic blood pressure; CAD: coronary artery disease; BMI: body mass index; WC: waist circumstance; HR: heart rate; FBG: fasting blood glucose; PBG: postprandial blood glucose; HDL-c: high-density lipoprotein-cholesterol; LDL-c: low-density lipoprotein-cholesterol; ACEI/ARB: angiotensin converting enzyme inhibitor/angiotensin receptor blocker.

**Figure 1:**
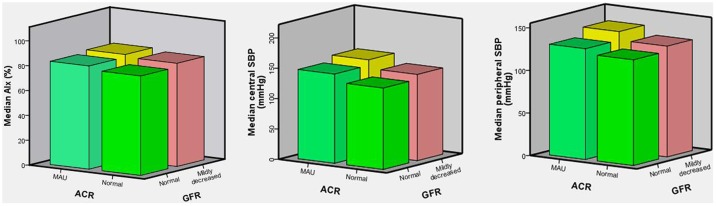
AIx and BP of the study population categorized into 4 subgroups based on the presence or absence (normal GFR) of mildly decreased GFR and the presence or absence (normal ACR) of MAU. The difference between subgroups was highly significant (all p<0.001). **Abbreviation**: AIx: augmentation index; BP: blood pressure; GFR: glomerular filtration rate; ACR: albumin-to-creatinine ratio; MAU: microalbuminuria; SBP: systolic blood pressure.

The description of AIx and BP in the four subgroups is displayed in Table 2. After participants were categorized into four subgroups based on the presence or absence of MAU and mildly decreased GFR, not only pSBP, but also AIx and cSBP, progressively increased from the subgroup without both of MAU and mildly decreased GFR to the subgroups with either one of them, and arrived at top in the subgroup with both of them (p<0.001 for all, Figure 1). Compared with the reference category without MAU and mildly decreased GFR, the OR values significantly increased from the category with either one of MAU and mildly decreased GFR to the category with both of them (p<0.001 for all). The bivariate correlation of UACR and GFR with other variables is listed in Table 3. The AIx, cSBP and pSBP were significantly correlated with UACR and GFR (P<0.001 for all).

**Table 2:** Description of AIx and BP in subgroups based on the presence or absence of MAU and mildly decreased GFR.

Variables	Normal UACRand GFR	Normal UACR andmildly decreasedGFR	MAU and normalGFR	MAU and mildlydecreased GFR	P value
cSBP (mmHg)	134(121–148)	142(130–158)	147(131–167)	156(139–174)	<0.001
OR value (95%CI)	Reference	1.018(1.013–1.023)	1.029(1.023–1.035)	1.039(1.032–1.046)	
P value		<0.001	<0.001	<0.001	
AIx (%)	80(72–88)	83(76–89)	83(78–91)	85(76–91)	<0.001
OR value (95%CI)	Reference	1.021(1.011–1.030)	1.028(1.016–1.040)	1.031(1.016–1.046)	
P value		<0.001	<0.001	<0.001	
pSBP (mmHg)	124(112–140)	130(120–144)	130(120–150)	140(125–160)	<0.001
OR value (95%CI)	Reference	1.018(1.013–1.024)	1.030(1.023–1.037)	1.045(1.037–1.053)	
P value		<0.001	<0.001	<0.001	

**Abbreviation**: AIx: augmentation index; BP: blood pressure; MAU: microalbuminuria; GFR: glomerular filtration rate; UACR: uric albumin-to-creatinine ratio; cSBP: central systolic blood pressure; OR: odd ratio; CI: confidence interval; pSBP: peripheral systolic blood pressure.

**Table 3:** Bivariate correlation of UACR and GFR in the whole cohort.

Variables	UACR		GFR	
	Correlationcoefficient	P value	Correlationcoefficient	P value
Main variables				
cSBP (mmHg)	0.269	<0.001	−0.241	<0.001
AIx (%)	0.164	<0.001	−0.116	<0.001
pSBP (mmHg)	0.213	<0.001	−0.196	<0.001
Baseline variables				
Age (year)	0.168	<0.001	−0.508	<0.001
Males (%)	−0.129	<0.001	0.043	0.052
Smoking (%)	−0.100	<0.001	−0.042	0.060
CAD (%)	−0.026	0.248	−0.024	0.285
Type 2 diabetes (%)	0.124	<0.001	−0.067	0.002
BMI (kg/m^2^)	0.111	<0.001	−0.136	<0.001
WC (cm)	0.032	0.148	−0.194	<0.001
HR (bpm)	0.084	<0.001	0.049	0.026
FBG (mmol/L)	0.121	<0.001	−0.121	<0.001
PBG (mmol/L)	0.163	<0.001	−0.163	<0.001
Triglyceride (mmol/L)	0.129	<0.001	−0.163	<0.001
HDL-c (mmol/L)	−0.017	0.437	−0.052	0.020
LDL-c (mmol/L)	0.085	<0.001	−0.204	<0.001
ACEI/ARB (%)	0.040	0.072	−0.044	0.046
Statins (%)	0.005	0.811	−0.009	0.685
Dependent variables				
UACR (mg/g)	1.000	1.000	−0.017	0.436
GFR (ml/min/1.73 m^2^)	−0.017	0.436	1.000	1.000

**Abbreviation**: UACR: uric albumin-to-creatinine ratio; GFR: glomerular filtration rate; cSBP: central systolic blood pressure; AIx: augmentation index; pSBP: peripheral systolic blood pressure; CAD: coronary artery disease; BMI: body mass index; WC: waist circumstance; HR: heart rate; FBG: fasting blood glucose; PBG: postprandial blood glucose; HDL-c: high-density lipoprotein-cholesterol; LDL-c: low-density lipoprotein-cholesterol; ACEI/ARB: angiotensin converting enzyme inhibitor/angiotensin receptor blocker.

The independent association of AIx and BP with MAU and mildly decreased GFR is shown in Table 4. The association of AIx, cSBP and pSBP with MAU and mildly decreased GFR was highly significant in the first model, and held even after adjustment in the second and third models (P<0.05 for all); meanwhile, the association strength of MAU and mildly decreased GFR with cBP was similar to those with pBP in the first, second and third models.

**Table 4:** Independent association of MAU and mildly decreased GFR with AIx and BP according to multivariate analysis.

Variables	Models	MAU		Mildly decreased GFR	
		OR value (95%CI)	P value	OR value (95%CI)	P value
cSBP (mmHg)	1st model^a^	1.026(1.021–1.030)	<0.001	1.016(1.012–1.020)	<0.001
	2nd model^b^	1.025(1.019–1.030)	<0.001	1.016(1.012–1.020)	<0.001
	3rd model^c^	1.024(1.018–1.029)	<0.001	1.011(1.007–1.016)	<0.001
AIx (%)	1st model^a^	1.022(1.013–1.032)	<0.001	1.017(1.009–1.025)	<0.001
	2nd model^b^	1.013(1.003–1.024)	0.014	1.016(1.008–1.025)	<0.001
	3rd model^c^	1.015(1.004–1.026)	0.009	1.012(1.003–1.021)	0.008
pSBP (mmHg)	1st model^a^	1.023(1.018–1.028)	<0.001	1.017(1.012–1.022)	<0.001
	2nd model^b^	1.021(1.016–1.027)	<0.001	1.018(1.013–1.022)	<0.001
	3rd model^c^	1.018(1.012–1.024)	<0.001	1.013(1.008–1.018)	<0.001

**Note**: ^a^First model: no adjusted; ^b^Second model: regression model adjusted by age and gender; ^c^Third model: regression model adjusted by age, gender, smoking, coronary artery disease, type 2 diabetes, body mass index, waist circumstance, heart rate, fasting blood glucose, postprandial blood glucose, triglyceride, high-density lipoprotein-cholesterol, low-density lipoprotein-cholesterol, angiotensin converting enzyme inhibitor/angiotensin receptor blocker, and statins use.

**Abbreviation**: MAU: microalbuminuria; GFR: glomerular filtration rate; AIx: augmentation index; BP: blood pressure; OR: odd ratio; CI: confidence interval; cSBP: central systolic blood pressure; pSBP: peripheral systolic blood pressure.

## Discussion

The current analysis presents three main findings concerning the Chinese community-dwelling population. First, there was a high prevalence of MAU and mildly decreased GFR. Second, AIx, cBP and pBP were independently associated with MAU and mildly decreased GFR. Third, cBP did not exhibit stronger association with MAU and mildly decreased GFR compared with pBP. To the best of our knowledge, this study is the first report to explore the association between AIx, cBP and pBP with MAU and mildly decreased GFR in Chinese community-dwelling population.

The relationship of renal demage with arterial compliance evaluated by AIx and blood pressure including cBP and pBP has caught much attention recently. Previous studies have examined the association of AIx, cBP and pBP with GFR and UACR as a continuous variable, but the results remained controversial [1]–[10]. Meanwhile, in spite of existing several studies concerning relationship of AIx, cBP and pBP with CKD, macroabuminuria and ESRD [3], [11]–[13], the association of AIx, cBP and pBP with mildly decreased GFR and MAU has not been fully investigated, and no clear conclusion could be drawn from the available data [3], [6], [9], [14], [15]. In particular, most studies have paid attention to the correlation of AIx, cBP and pBP with CKD and ESRD, but their relation to the early stage of renal function, i.e., mildly decreased GFR, is a relatively neglected topic [15]. The study by Dr. Ashish Upadhyay has manifested that MAU was related to cBP but not AIx [3]. However, Dr. Costas Tsioufis has verified that MAU was significantly correlated with AIx [6]. Our current analysis demonstrated the independent relationship of AIx, cBP and pBP with MAU and mildly decreased GFR. It is possible that increased blood pressure and decreased arterial compliance, from any cause, lead to MAU and mildly decreased GFR by afferent arteriolar barotraumas; it is also possible that MAU and mildly decreased GFR, from any cause, induce increased blood pressure and decreased arterial compliance [20]. Lately, Dr Junichiro Hashimoto has reported that increased blood pressure caused renal microvascular damage through altered renal hemodynamics resulting from increased peripheral resistance and/or increased flow pulsation [21]. In addition to afferent arteriolar barotraumas, because blood pressure and arterial compliance are closely related to generalized endothelial dysfunction and subclinical atherosclerosis, these latter might represent other pathophysiological mechanisms through which blood pressure and arterial compliance lead to early signs of renal damage [22]–[24]. On the other hand, renal function has been shown to be a major determinant of accelerated progression of arterial stiffness in clinical studies [25]. In animal models, the accumulation of collagen, advanced glycosylation end products, and asymmetric dimethylarginine in the arterial wall as well as oxidative stress might negatively influence the distensibility of the arterial wall [26], [27]. Change in water and salt balance [28], leading to renin-angiotensin-aldosterone system activation, might stimulate the accumulation of collagen instead of elastin; collagen represents the more rigid component of the arterial wall [29]. Thus, the change of renal function might be determined by not only blood pressure level, but also arterial compliance.

Limited studies have compared the association strength of cBP and pBP with renal demage. Dr. Kang-Ling Wang has reported that cBP had a stronger association with GFR than pBP [16]. Dr. Ulf Neisius has demonstrated that the correlation of cBP with UACR was very close to that of pBP with UACR [17]. The lastest study from Dr. Anna Oliveras has found that cBP and pBP were similarly associated with MAU [9]. The finding in the current analysis confirmed that there was insignificant difference between the relationship of cBP and pBP with MAU and mildly decreased GFR. Due to its inconvenience and high cost, measurement of cBP has not been widely used in clinical practice and community survey up to now. If indeed cBP has no closer association with MAU and mildly decreased GFR than pBP, it is no doubt that pBP is a better choice to identify these early renal damage in clinical practice and community survey.

Several limitations in our study should be noted. First, the Chinese modified MDRD equation is based on the MDRD equation, which has been not only proved to be accurate and valid in general population, but also most commonly used as the GFR estimating equation. However, it tends to underestimate the GFR levels in the mildly decreased GFR range and thus increase the numbers of participants with mildly decreased GFR. Second, in order to avoid the disgust of study participants and the inaccurate data from study participants, we did not talk about the socioeconomic status in our analysis.

## Conclusions

The current analysis demonstrates the high prevalence of MA and mildly decreased GFR in Chinese community-dwelling population, and shows that MAU and mildly decreased GFR are independently related to not only pBP, but also cBP and AIx. However, cBP did not exhibit stronger association with MAU and mildly decreased GFR compared with pBP. Consideration of the inconvenience and high cost of measuring cBP, the measurement of pBP could still be applied in the prevention, identification and treatment of MA and mildly decreased GFR in Chinese community-dwelling population, which has garnered increased attention in recent years.
